# Effect of Modification on the Fluid Diffusion Coefficient in Silica Nanochannels

**DOI:** 10.3390/molecules26134030

**Published:** 2021-07-01

**Authors:** Gengbiao Chen, Zhiwen Liu

**Affiliations:** College of Automotive and Mechanical Engineering, Changsha University of Science and Technology, Changsha 410114, China; 20203030594@stu.csust.edu.cn

**Keywords:** porous silica gel, hydroxylation, silylation, wall characteristics, diffusion coefficient

## Abstract

The diffusion behavior of fluid water in nanochannels with hydroxylation of silica gel and silanization of different modified chain lengths was simulated by the equilibrium molecular dynamics method. The diffusion coefficient of fluid water was calculated by the Einstein method and the Green–Kubo method, so as to analyze the change rule between the modification degree of nanochannels and the diffusion coefficient of fluid water. The results showed that the diffusion coefficient of fluid water increased with the length of the modified chain. The average diffusion coefficient of fluid water in the hydroxylated nanochannels was 8.01% of the bulk water diffusion coefficient, and the diffusion coefficients of fluid water in the –(CH_2_)_3_CH_3_, –(CH_2_)_7_CH_3_, and –(CH_2_)_11_CH_3_ nanochannels were 44.10%, 49.72%, and 53.80% of the diffusion coefficients of bulk water, respectively. In the above four wall characteristic models, the diffusion coefficients in the *z* direction were smaller than those in the other directions. However, with an increase in the silylation degree, the increased self-diffusion coefficient due to the surface effect could basically offset the decreased self-diffusion coefficient owing to the scale effect. In the four nanochannels, when the local diffusion coefficient of fluid water was in the range of 8 Å close to the wall, Dz was greater than Dxy, and beyond the range of 8 Å of the wall, the Dz was smaller than Dxy.

## 1. Introduction

Recently, fluid transport properties in nanochannels have attracted extensive attention [1,2,3]. Porous silica gel [4] has been widely used in drug delivery [5], vibration reduction [6], and other aspects due to its internal nanoporous structure and mature preparation technology. 

Compared with macroscopic flow, self-diffusion of the fluid in micro-nanochannels is significantly different. The “surface effect” between the fluid and wall molecules and the “scale effect” in the confined space are the main factors influencing the self-diffusion behavior of the fluid in micro-nanochannels. Many scholars have carried out multiple simulation calculation studies on the scale effect. Magda et al. [7] found that the diffusion coefficient of water molecules was correlated with the average density during the transmission process in nanochannels, and when the channel diameter was more than 10 times the diameter of water molecules, the average diffusion coefficient of water in the channels was similar to that of bulk water. Bourg et al. [8] simulated and observed that there were three density statistical layers in the silica nanochannels with diameters of 2 and 4 nm, but not in the silica nanochannels with a diameter of 1 nm. The diffusion coefficient of fluid water increased with the channel diameter. The first layer of fluid water near the channel wall was relatively stable, and it was less affected by water in the other areas of the channel. Fasano et al. [9] simulated the diffusion behavior of water molecules in carbon nanotube arrays, and they found that the diffusion coefficient of fluid water was related to the size of the nanochannels and the change in the diffusion coefficient of water molecules could be controlled by inducing an electric field. Liu et al. [10] investigated the self-diffusion coefficient of water in different directions, and they reported that the self-diffusion coefficient of water along the channel direction was about 4–5 times that of water along the pore direction.

There are some reports on the influence of surface effects on the fluid diffusion behavior in micro-nanochannels. For example, Zhao et al. [11] found that the diffusion capacity of water spontaneously entering the original graphene was 1–2 orders of magnitude higher than that of water entering the hydroxylated graphene. Wei et al. [1] reported that titanium dioxide (TiO_2_) nanochannels were hydrophilic, and the liquid water formed hydrogen bonds with the TiO_2_ wall surface in the channels. The surface of the carbon-covered channels was hydrophobic, but it enhanced the diffusion characteristics of the water molecules. Fasano et al. [12] also studied the influence of hydrophilicity and hydrophobicity on the diffusion characteristics. It was found that the self-diffusion coefficient of water in hydrophobic zeolites with the hydrophilic group defect decreased with increased hydrophilic groups. 

Currently, there are few reports on the diffusion characteristics of the fluid in modified silica nanochannels. In this study, the equilibrium molecular dynamics method was used to simulate the self-diffusion behavior of water in the hydroxylated and silanized silica channels with different chain lengths. A qualitative description of the relationship between the number of silanized chains on the channel wall and the diffusion coefficient of the fluid water was obtained. The diffusion behavior described in this paper refers to the dynamic self-diffusion behavior of fluid molecules under the action of molecular thermal motion without a concentration gradient. The water molecules follow the Fickian diffusion mechanism [13], where the mean square displacement of water molecules is proportional to time and the diffusion ability is evaluated by the diffusion coefficient.

## 2. Nanoflow Model

### 2.1. Modeling

**Figure 1:**
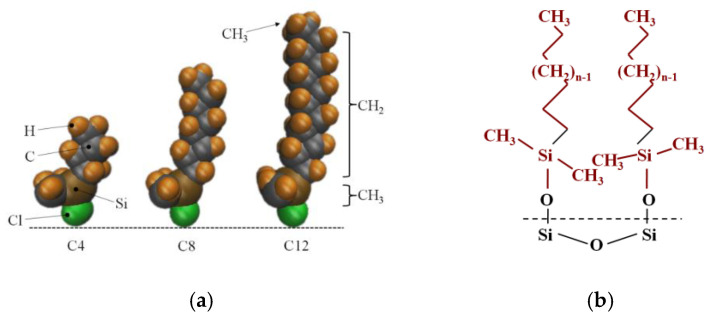
The process diagram of modified silica gel. (**a**) Alkyl chlorosilane molecule; (**b**) the molecular structure of the nanochannel wall after modifying.

Porous silica is a compound comprising amorphous silicon dioxide whose unmodified silica surface contains hydroxyl –OH groups, modified hydrophobic silicone is a porous hydrophobic silicone compound formed by porous silicone particles modified by linear chains of alky chlorosilanes, ClSi (CH_3_)_2_C_n_H_2n−1_. After the silicone is silanized, hydroxyl –OH on the surface is replaced by a linear chain of silanyl (–Si(CH_3_)_2_C_n_H_2n+1_), Si in the silane baseline linear chain is consistent with Si in amorphous silica, and the difference in modification degree ultimately leads to the difference in chain length n. In order to facilitate the study, the molecular composition of the contact interface characteristics of the nanochannel modified layer is regarded as an alkyl baseline linear chain composed of carbon and hydrogen atoms (-C_n_H_2n+1_, characterized by n for chain length). The modification process is shown in Figure 1.

**Figure 2:**
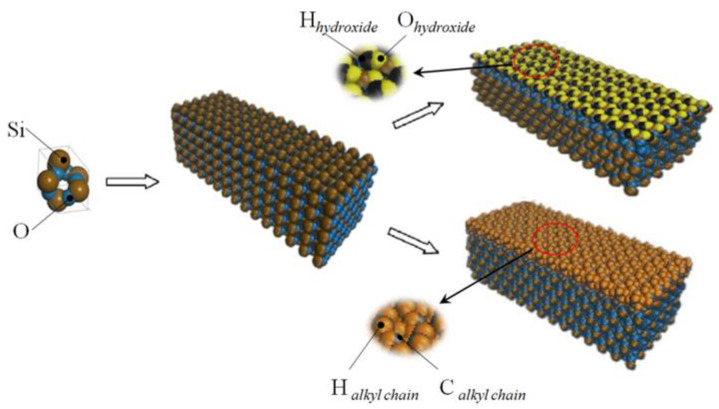
The modeling process diagram of the nanochannel wall.

**Figure 3:**
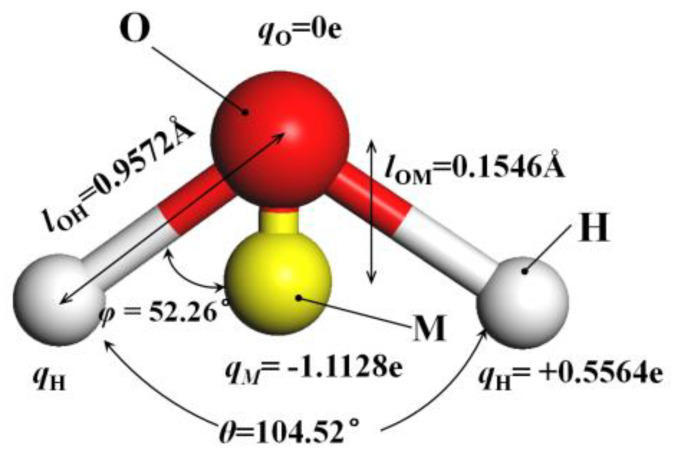
The model of TIP4P/2005 water molecular.

**Figure 4:**
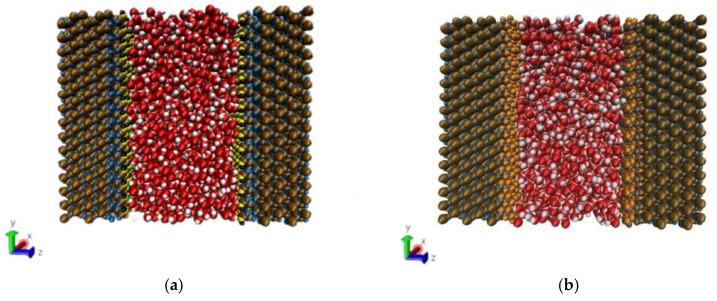
Micro-flow system in a nanochannel: (**a**) hydroxylated nanochannel; (**b**) silanized nanochannel. Color legend: silicon Si (ochre), SiO_2_ oxygens O_SiO2_ (blue), hydroxide oxygens O_hy_ (yellow), and hydroxide hydrogens H_hy_ (black), alkyl chain C (_ch2/ch3_) (gray), alkyl chain H (_ch2/ch3_) (orange), water O_w_ (red), water H_w_ (white).

The method of building the model [14] was as follows in Figure 2: the wall surface of the nanochannels comprised α-SiO_2_, and its protocells expanded into a 5 × 6 × 15 super crystal package along with the *x*, *y*, and *z* directions, namely the wall surface of the channels. Then, the surface was modified with –OH, –(CH_2_)_3_CH_3_, –(CH_2_)_7_CH_3_, and –(CH_2_)_11_CH_3_, with a modification rate of surface silylation of 50%, it means that 50% of the Si atoms in the entire nanochannel wall are modified, and the surface of the nanochannel wall is completely alkyl modified. To study the surface effect of the modified layer more effectively, the circular hole channel was simplified as a two-plate flow channel. The TIP4P/2005 model [15] was used for water molecules in Figure 3, The channel widths of the four mock systems are all 40 Å, and the calculated fluid zone is 32.28 × 73.90 × 40.00 Å^3^. The initial density of fluid water in the channel is 1g/cm^3^, it contains a total of 2632 water molecules and the flow model of nanochannels is shown in Figure 4.

### 2.2. Force Field Analysis

The multiphase system mainly comprised α-SiO_2_, the surface hydroxyl group –OH or the alkyl linear chain (–C_n_H_2n + 1_), and water molecule (H_2_O). Total potential energy function between atoms is expressed as follows:

(1) $$U=\underset{\mathrm{bond}}{\sum }u_{b}\left(l\right)+\underset{\mathrm{angle}}{\sum }u_{\theta }\left(\theta \right)+\underset{\mathrm{dihedral}}{\sum }u_{\omega }\left(\omega \right)+\underset{\mathrm{out}-\mathrm{of}-\mathrm{plane}}{\sum }u_{\chi }\left(\chi \right)+\underset{\mathrm{cross}}{\sum }u\left(l,\;\theta ,\;\omega \right)+U_{Coulomb}+U_{LJ}$$

where

(2) $$U_{LJ}=\overset{N}{\underset{i=1,\;j>1}{\sum }}4\varepsilon_{ij}\left[{\left(\frac{\sigma_{ij}}{r_{ij}}\right)}^{12}-{\left(\frac{\sigma_{ij}}{r_{ij}}\right)}^{6}\right]$$

(3) $$U_{Coulomb}=\overset{N}{\underset{i=1,\;j>1}{\sum }}\frac{q_{i}q_{j}}{4\pi \varepsilon_{0}r_{ij}}\;\;\;$$

where *θ*, *l*, *ω*, and *χ* represent the bond angle, bond length, dihedral angle and vibration angle from the plane, respectively. *N* represents the total number of atoms. *r_ij_* represents the distance from the ith atom to the jth one. *ε_ij_* and *σ_ij_* are Lennard-Jones (LJ) functional potential energy wells and distance parameter, respectively. *q_i_* indicates the quantity of electric charge of atom *i*, and *ε*_0_ is permittivity of vacuum. The particle–particle and particle–mesh (PPPM) algorithm [16] was used to calculate the long-range Coulomb effect. The selection of the LJ potential energy parameter, bond, and charge quantity of silica and modified alkane chain fit according to the experimental data, which is shown in Table 1 [14,17,18,19].

### 2.3. Simulation Details

The self-diffusion behavior of fluid water in silica gel nanochannels with surface groups, such as –OH, –(CH_2_)_3_CH_3_, –(CH_2_)_7_CH_3_, and –(CH_2_)_11_CH_3_, was studied. The self-diffusion parameters of fluid water in silica gel nanochannels were simulated by the equilibrium molecular dynamics method. The four systems were simulated at standard atmospheric pressure and temperature T = 298 K, using the Large-scale Atomic/Molecular Massively Parallel Simulator (LAMMPS) software to immobilize the channel wall atoms. The SHAKE algorithm was selected to limit the bond length and angle of water molecules. The velocity-Verlet algorithm was used to solve the motion equation of particles with a time step of 0.1 fs, and system relaxation time of 15 ps reached the initial equilibrium state using the NPT ensemble. Then, the system was run under the NVE ensemble for 20 ps. During this period, physical parameters, such as mean square displacement and diffusion coefficient of fluid water, were calculated to analyze the influence of modified convective water on the transport characteristics of silica nanochannels.

## 3. Results and Analysis

### 3.1. Simulation of Diffusion Coefficients of Fluids in Nanochannels

According to the fluctuation-dissipation theory of non-equilibrium statistical thermodynamics, the Einstein method and the Green–Kubo method can be used to simulate the diffusion coefficient of the fluid in nanochannels.

The Einstein method obtains the diffusion coefficient by calculating the mean square displacement of fluid:

(4) $$MSD_{i}\left(t\right)=\langle {\left|\vec{r_{i}}\left(t\right)-\vec{r_{i}}\left(0\right)\right|}^{2}\rangle \;(i=x,y,z)$$

(5) $$D_{MSD}=\underset{t\to \infty }{\lim}\frac{1}{2nt}\langle {\left|\vec{r_{i}}\left(t\right)-\vec{r_{i}}\left(0\right)\right|}^{2}\rangle$$

Equation (4) is the mean square displacement (*MSD*) equation, where *r (t)* represents position of the water molecular mass center at time *t* and <> represents the average value of displacement of all water molecules in the system from initial time to time *t*. and Equation (5) indicates the diffusion coefficient obtained by calculating the time variability of *MSD* at that time by the Einstein relation. n represents the calculated spatial dimension, for example, *n* = 3 indicates the diffusion coefficient in three-dimensional space. 

**Figure 5:**
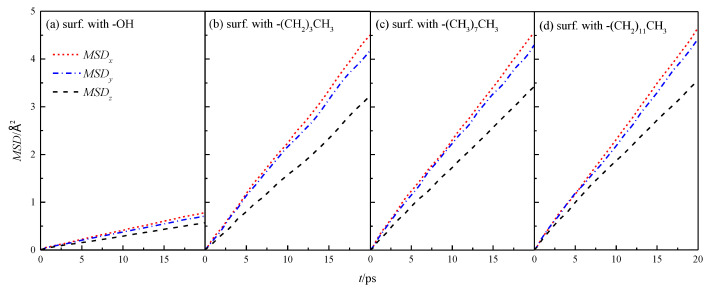
*MSD* in the *x*, *y*, and *z* directions of water in a nanochannel with different surface characteristics.

The *MSD* of fluid water in the four types of wall characteristic nanochannels in *x*, *y*, and *z* directions was simulated by Equation (4), as shown in Figure 5. 

**Figure 6:**
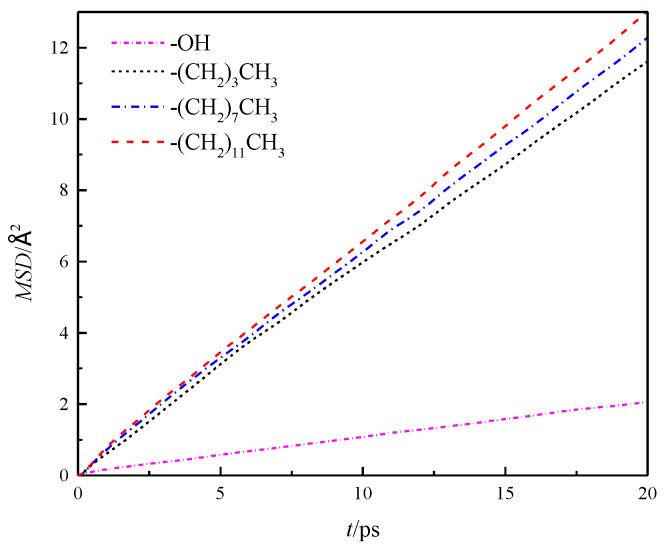
Overall average *MSD* of water in a nanochannel with different surface characteristics.

The mean azimuthal displacement of fluid water in the channels increased gradually in the three directions over time, and the mean azimuthal displacement of fluid water in *x* and *y* directions was approximately the same, but it was greater than that in the *z* direction. This finding was similar to the results presented in the study cited in reference [8], which showed that the *z* direction was the aperture direction. Its average azimuthal displacement was smaller than the channel direction limited by the wall. To compare the results of different wall characteristics, *MSD* of the walls in four channels was calculated further, as shown in Figure 6. The results showed that the overall average *MSD* of the fluid water in the silanized channels was greater than that in the hydroxylated channels, and the *MSD* of the fluid water in the channels gradually increased with the length of the silanization-modified chain.

**Figure 7:**
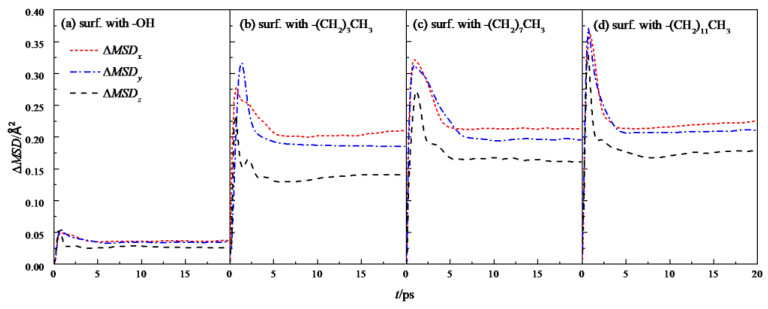
Δ*_MSD_* in the *x*, *y*, and *z* directions of water in a nanochannel with different surface characteristics.

**Figure 8:**
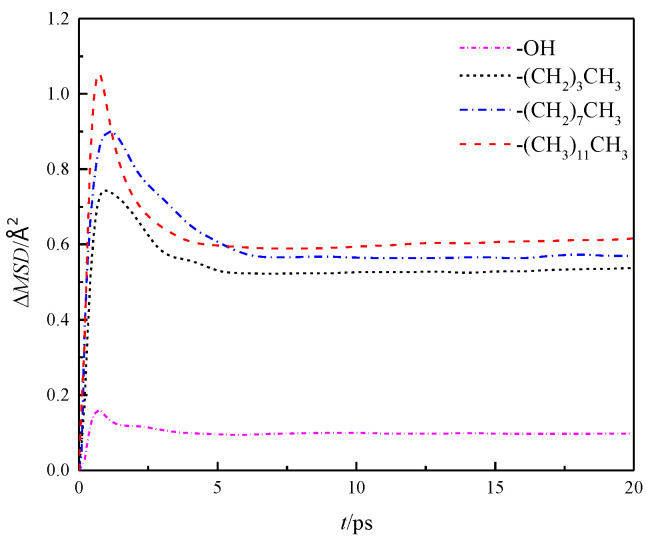
Overall average Δ*MSD* of water in nanochannel with different surface characteristics.

Further analysis was made on the mean square displacement rate (Δ*MSD*) of fluid water in the four wall features of the silicon dioxide nanochannel. By looking at the first derivative of the mean square displacement curve function, it is obtained that the $MSD_{x\backslash y\backslash z}$ in each nanochannel is shown in the three directions of *x*, *y* and *z*, as shown in Figure 7, and the overall average Δ*MSD* is shown in Figure 8.

According to the above result of the mean square displacement rate, using the variation Form (6) of the Einstein relation, the self-diffusion coefficients (*D_MSD_*) of fluid water in the *x*, *y* and *z* directions in the four types of wall characteristic channels were calculated respectively, and then the total average diffusion coefficient of fluid water in the nanochannels was calculated by Equation (7). The calculation results are shown in Table 2.

(6) $$D_{MSD\left(i\right)}=\underset{t\to \infty }{\lim}\frac{MSD\left(i\right)}{2t}=\underset{t\to \infty }{\lim}\frac{\langle {\left|\vec{r_{i}}\left(t\right)-\vec{r_{i}}\left(0\right)\right|}^{2}\rangle }{2t}\;i=(x,y,z)$$

(7) $$D_{MSD}=\frac{D_{MSD\left(x\right)}+D_{MSD\left(y\right)}+D_{MSD\left(z\right)}}{3}$$

**Figure 9:**
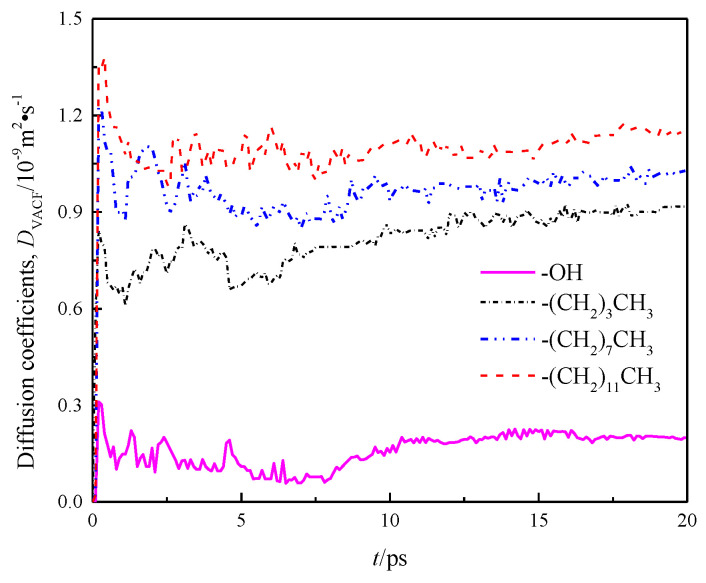
Diffusion coefficient *D_VACF_* of water in a nanochannel with different surface characteristics.

To verify the correctness of the results, control simulation was introduced and the self-diffusion coefficient *D_VACF_* was obtained by integrating the velocity auto-correlation function (VACF) of fluid water in the nanochannels with the Green–Kubo method (8).

(8) $$D_{VACF}=\frac{1}{3}\int_{0}^{\infty }dt\langle \vec{v}\left(t\right)-\vec{v}\left(0\right)\rangle$$

where, *v*(*t*) is the speed of mass center of water molecule at time *t*, and <> indicates all water molecules in the system from initial time to time *t*. *D_VACF_* is the diffusion coefficient obtained by calculating the change rate of particle velocity over time through the Green–Kubo relation. The calculation result is shown in Figure 9. 

The diffusion coefficients of each channel wall calculated by the Einstein method and the Green–Kubo method, as well as the diffusion coefficients in *x*, *y*, and *z* directions are listed in Table 2. 

A comparison of *D_MSD_* and *D_VACF_* among the four models indicates basically same calculation results of the Einstein and Green–Kubo methods, suggesting that the data are reliable. Meanwhile, Table 2 demonstrates that there are obvious differences in the internal diffusion coefficient of fluid water in different wall features of silicon dioxide nanochannels, the diffusion coefficients in the *x*, *y* and *z* directions of each channel are also unequal, as shown by anisotropy, and the diffusion coefficients in the *z* direction of all models were smaller than those in *x* and *y* directions due to the limitation of wall space. Because the circular channel model was simplified as a two parallel plate model [20] in this study, the diffusion coefficient of fluid water in the *z* direction was limited, while the diffusion coefficients of fluid water in *x* and *y* directions were basically the same. Owing to the scale effect, the total diffusion coefficients of fluid water in the four models were smaller than the total diffusion coefficients of bulk water D_EXP_ = 2.26~2.29 × 10^−9^ m^2^ · s^−1^ [21].

The self-diffusion coefficient of fluid water in a hydroxylated channel was only about 1/5 of the self-diffusion coefficients in the other three models on comparing the four channel walls with different surface characteristics. This was because hydroxyl is a hydrophilic group and it forms hydrogen bonds with water, which hinders the self-diffusion behavior of water and reduces the self-diffusion coefficient of water. The mean square displacement of the modified nanochannel is proportional to the hydrophobic strength. At the same time, the self-diffusion coefficient of fluid water in the *z* direction gradually increased with the length of the silane-modified chain and the growth rate was 20–25%. 

### 3.2. The Radial Distribution Function and the Velocity Distribution of the Fluid

**Figure 10:**
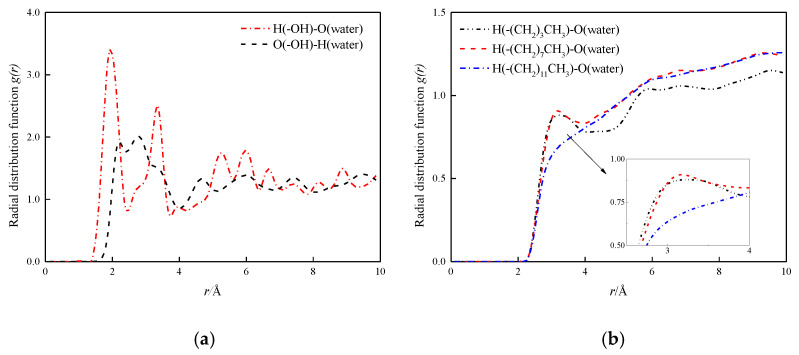
H-bonds radial distribution function at solid–liquid interface. (**a**) H-bonds radial distribution function at solid–liquid interface in hydroxylation nanochannel; (**b**) H-bonds radial distribution function at solid–liquid interface in silanized nanochannel.

In order to study the role of hydrogen bonds in depth, the radial distribution function (RDF, g(r)) of the hydrogen bond between the fluid and the channel wall in the four mock systems is analyzed and discussed. First, the radial distribution functions between hydroxyl groups in hydroxyl surface and O and H atoms in water molecules are calculated, as shown in Figure 10a. The first peak of RDF between H and O in the hydromolecular wall is at 1.77 Å, indicating a strong hydrogen bond between the two [22]. The first peak of O and H in the water in the wall is at 2.16 Å, which is beyond the distance from which the hydrogen bond is formed. In Figure 10b, the radial distribution function between H and O in water in the modified wall is shown, wherein the carbon chain length of the modified layer is –(CH_2_)_3_CH_3_, –(CH_2_)_7_CH_3_ and –(CH_2_)_11_CH_3_. The results show that the surface of the modified layer carbon chain length –(CH_2_)_3_CH_3_ and –(CH_2_)_7_CH_3_ has small peak fluctuations at 3.30 Å and 3.15 Å, both distances exceed the distance required to form a hydrogen bond, indicating the formation of hydrogen-free bonds between water molecules and modified silanized walls, respectively, the peak of the surface of –(CH_2_)_11_CH_3_ basically disappears here, indicating the interaction between the two is characterized by weak coulomb and Van der Waal, and decreases as the chain length increases.

By comparing the radial distribution function between different characteristics wall surfaces and water molecules, the following conclusions can be obtained: hydroxylized wall surface and water molecules have a strong hydrogen bonding effect, which is also one of the reasons for its hydrophilicity, and the formation of hydrogen-free bonds between modified silanized wall surfaces and water molecules, so that the moisturizing of nanochannels is shown as strong hydrophobic. This also reasonably explains the positive relationship between diffusion coefficient and hydrophobic strength.

**Figure 11:**
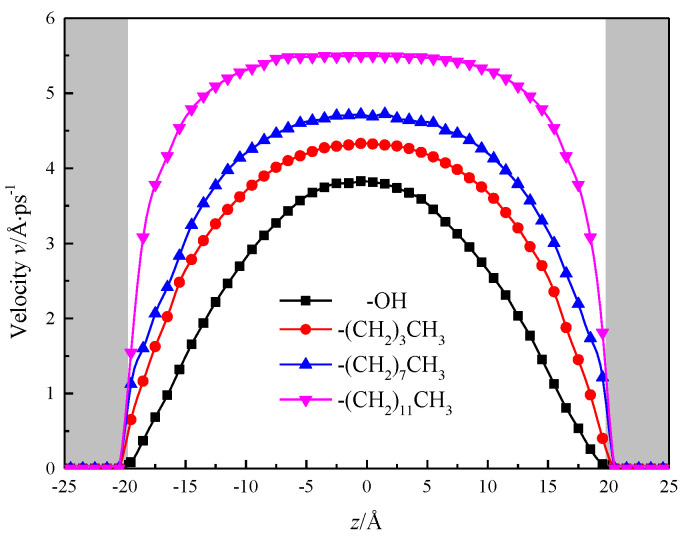
Velocity distribution of water in nanochannel with different surface characteristics.

Calculated by simulation, the fluid area is divided into 40 layers in the direction of *z*, each with a thickness of 1 Å, after the simulation system has reached equilibrium, the velocity distribution of fluids in the nanochannel is counted as shown in Figure 11, and the gray shadows in the figure represent the channel wall surface in order to visually observe the velocity distribution of fluid water within the nanochannel. To be observed after the system reaches equilibrium, the velocity distribution of fluid water in the nanochannel is similar to its macro-flow, and its velocity distribution curve is parabolic. However, due to the solid–liquid potential and the characteristic molecular layer of the channel wall, there is a stable viscosity layer at the solid–liquid interface in the hydroxylized nanochannel, the fluid molecules are distributed in an orderly manner, and the viscosity of the fluid increases, the shear rate becomes smaller, no velocity slip, nanochannel The performance is strong hydrophilic, while the silanized nanochannel solid liquid interface with the enhancement of the degree of modification, fluid viscosity gradually decreased, shear rate gradually become larger, the phenomenon of “speed slip”, as a strong hydrophobic.

At the center of the nanochannel, the calculated statistics show that the fluid velocity in the hydroxylized nanochannel is 3.82 Å/ps, while the alkane chain is –(CH_2_)_3_CH_3_, –(CH_2_)_7_CH_3_, –(CH_2_)_11_CH_3_ silane, the fluid flow rate in the nanochannel is 4.33 Å/ps, 4.70 Å/ps, 5.49 Å/ps, and the results show that the fluid flow rate is positively correlated with the growth of alkyl chain, and the hydrophobicity of nanochannel is enhanced, which is beneficial to increase the flow of the channel. The velocity distribution of fluid water in the channel with alkyl chain length of –(CH_2_)_11_CH_3_ is close to the nature of macrofluids [23,24,25].

### 3.3. Simulation of Local Diffusion Coefficients of Fluids in Nanochannels

In order to further explore the local diffusion coefficient of fluid water in its nanochannel before and after silicone modification, the fluid is divided along the fluid area parallel to the *x*–*y* plane into a 2 Å thick flow layer with a total of 20 layers. Because the nanochannel is a symmetrical channel in the direction of *z*, it is possible to calculate and analyze the 10 layers of fluid layer on the inside side of the nanochannel.

Since nanochannels limit the z-velocity of fluid molecules near the wall, the velocity autocorrelation function integral of the tropospheric water in the nanochannel is used to obtain local diffusion coefficients [26]. Therefore, the local diffusion coefficients *D_z_* and *D_xy_*, which are parallel and perpendicular to each stratosphere of the channel wall, are calculated and analyzed by Einstein method:

(9) $$D_{z}=\langle \Delta_{z}^{2}\left(t\right)\rangle /\left(2t\right)$$

(10) $$D_{xy}=\langle \Delta_{x}^{2}\left(t\right)+\Delta_{y}^{2}\left(t\right)\rangle /\left(4t\right)$$

where $\Delta_{x}^{2}\left(t\right)$, $\Delta_{y}^{2}\left(t\right)$, $\Delta_{z}^{2}\left(t\right)$ is the mean square displacement on the three dimensions of *x*, *y*, *z* within each stratosphere.

**Figure 12:**
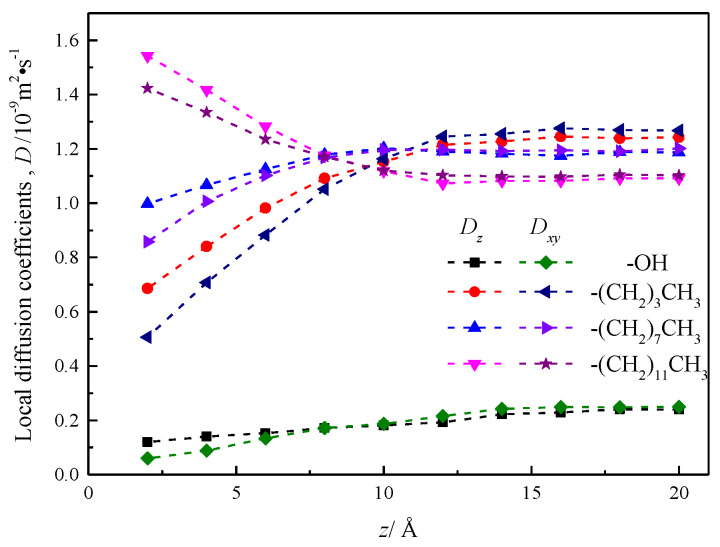
Local diffusion coefficient of water in nanochannel with different surface characteristics.

The results are shown in Figure 12, which shows that the local diffusion coefficient *D_z_* is greater than *D_xy_* in the range of 8 Å near the wall before and after the modification of the silica nanochannel, which is reasonably interpreted as water molecules entering the wall chain length or base group within the compartment, and beyond the distance range of 8 Å of the wall surface, the local diffusion coefficient of fluid water in the nanochannel is smaller than *D_xy_*, and the local diffusion coefficient of fluid water at the center of the nanochannel of silanized wall features is very different. The local diffusion coefficient curve of fluid water in the silicone nanochannel shows a certain smoothness, and does not show obvious stratification phenomenon like the local diffusion coefficient curve of water attached to the surface of hematite nanochannel [27].

The local diffusion coefficient of fluid water in the hydroxylization (–OH) nanochannel is smaller than its value in the silanized nanochannel, but with the increase of the distance between the flow layer and the wall surface, the local diffusion coefficient shows the trend of gradual increase.

## 4. Conclusions

In this study, the *MSD* and the self-diffusion coefficient of water in silica nanochannels without a concentration gradient were studied.

(1) Due to the influence of the scale effect, all the self-diffusion coefficients of fluid water in silica nanochannels were lower than the self-diffusion coefficients of bulk water, and the self-diffusion coefficients of –OH, –(CH_2_)_3_CH_3_, –(CH_2_)_7_CH_3_, and –(CH_2_)_11_CH_3_ were 8.01%, 44.10%, 49.72%, and 53.80% of the self-diffusion coefficients of bulk water, respectively. The diffusion coefficient of fluid water increased with the modification degree. In a sense, it is explained that before silicone modification, hydroxylized wall has a certain obstructive effect on the diffusion behavior of fluid water, while the modified silanized wall plays a certain role in promoting the diffusion behavior of fluid water.

(2) From the *MSD* in the *z* direction of –OH, –(CH_2_)_3_CH_3_, –(CH_2_)_7_CH_3_, and –(CH_2_)_11_CH_3_, the self-diffusion coefficient of silica nanochannels in the scale limited direction was much lower than that in the unrestricted direction. However, the surface effect of the wall features of the modified silicone nanochannel plays a leading role in the transmission of fluid water, the self-diffusion coefficient caused by the surface effect increased with the silane-modification degree, which basically offset the reduction in the self-diffusion coefficient caused by the scale effect.

(3) The hydroxylized channel wall and water molecules have a strong hydrogen bonding effect, making it appear hydrophilic. Regarding the modified silanized channel wall and water molecules hydrogen-free bond formation, the interaction between the two shows a weak coulomb force and Van der Waal force, reasonably revealing its hydrophobic mechanism.

(4) In the four nanochannels, *D_z_* is greater than *D_xy_* when the local diffusion coefficient of fluid water is in the range of 8 Å close to the wall, and beyond the range of 8 Å of the wall, the D_z_ is smaller than *D_xy_*. The local diffusion coefficient of fluid water near the distance of 8 Å. to the wall surface shows a nonlinear increasing trend in silicone nanochannels of –OH, –(CH_2_)_3_CH_3_, and –(CH_2_)_7_CH_3_, and is less than the local diffusion coefficient of fluid water at the center of the channel. The trend of fluid water in the silanized nanochannels characterized by wall surfaces –(CH_2_)_11_CH_3_ is the opposite, due to the diffusion behavior of wall-to-water molecules with a chain length of –(CH_2_)_11_CH_3_ and the mutually exclusive action of water molecules beyond the cohesion between water molecules.

## Figures and Tables

**Table 1:** The LJ potential energy parameters and coulombic charge in the model [12].

Composition of Multiphase System	Atomic Type	*ε*/eV	*σ*/Å	|e|
water	H_w_	0.0	0.0	0.5564
	O_w_	0.00803105	3.1589	−1.1128
hydroxyl	O_hy_	0.006595682	3.1538	−0.51
	H_hy_	0.001994750	0.4	0.32
silica	O_SiO2_	0.006595682	3.1538	−0.70
	Si	0.02602000	3.91996	bond incrementally
alky chain	C_ch2_	0.002862039	3.5	−0.148
	C_ch3_	0.002862039	3.5	−0.222
	H_ch2_	0.001140073	2.5	0.074
	H_ch3_	0.001300927	2.5	0.074

**Table 2:** Diffusion coefficient of water in nanochannel with different surface characteristics.

Nanochannel Type		–OH	–(CH_2_)_3_CH_3_	–(CH_2_)_7_CH_3_	–(CH_2_)_11_CH_3_
*_Diffusion coefficient_* (10^−9^ m^2^·s^−1^)	*x*	0.20	1.15	1.23	1.24
	*y*	0.19	1.06	1.17	1.21
	*z*	0.15	0.75	0.93	1.15
	*D_MSD_*	0.18	0.99	1.11	1.20
	*D_VACF_*	0.17	0.92	1.04	1.17

## Data Availability

The data presented in this study are available on request from the corresponding author.

## Sample Availability

Samples of the compounds are not available from the authors.
